# G-RCenterNet: Reinforced CenterNet for Robotic Arm Grasp Detection

**DOI:** 10.3390/s24248141

**Published:** 2024-12-20

**Authors:** Jimeng Bai, Guohua Cao

**Affiliations:** School of Mechanical and Electrical Engineering, Changchun University of Science and Technology, Changchun 130022, China; koko05773@163.com

**Keywords:** object detection, CenterNet, attention module search strategy, GSConv module

## Abstract

In industrial applications, robotic arm grasp detection tasks frequently suffer from inadequate accuracy and success rates, which result in reduced operational efficiency. Although existing methods have achieved some success, limitations remain in terms of detection accuracy, real-time performance, and generalization ability. To address these challenges, this paper proposes an enhanced grasp detection model, G-RCenterNet, based on the CenterNet framework. First, a channel and spatial attention mechanism is introduced to improve the network’s capability to extract target features, significantly enhancing grasp detection performance in complex backgrounds. Second, an efficient attention module search strategy is proposed to replace traditional fully connected layer structures, which not only increases detection accuracy but also reduces computational overhead. Additionally, the GSConv module is incorporated during the prediction decoding phase to accelerate inference speed while maintaining high accuracy, further improving real-time performance. Finally, ResNet50 is selected as the backbone network, and a custom loss function is designed specifically for grasp detection tasks, which significantly enhances the model’s ability to predict feasible grasp boxes. The proposed G-RCenterNet algorithm is embedded into a robotic grasping system, where a structured light depth camera captures target images, and the grasp detection network predicts the optimal grasp box. Experimental results based on the Cornell Grasp Dataset and real-world scenarios demonstrate that the G-RCenterNet model performs robustly in grasp detection tasks, achieving accurate and efficient target grasp detection suitable for practical applications.

## 1. Introduction

Robotic arms are essential for executing grasping tasks across various domains, such as service, healthcare, and agriculture. Therefore, advancements in robotic arm grasping technology play a pivotal role in driving the overall development of robotics [1,2]. The ability of a robotic arm to accurately perceive the grasp pose of a target object is crucial to ensure the successful execution of these tasks.

In current industrial applications, machine vision technology has been widely employed for object grasp detection due to its excellent performance and adaptability. Early research focused primarily on manually extracting feature information from image data to construct corresponding feature representations of target objects in unstructured environments [3]. This process involved mining and analyzing image data to extract key information related to grasping. Machine learning algorithms are then used to build mapping models between image features and grasp poses, enabling the automatic generation of grasp poses for unknown objects in unstructured settings. However, this method presents significant limitations in practical use. Since feature extraction relies heavily on manual input, it is deeply influenced by the researcher’s prior knowledge, leading to inconsistencies in feature extraction across different object types. In some cases, manually extracted features may fail to capture critical grasp information, adversely affecting the success rate and stability of the grasp [4].

The widespread application of deep learning in object detection has introduced new advancements in robotic arm grasp detection. By training deep neural networks, grasp detection systems can automatically identify and extract grasp-related features without relying on manually set prior knowledge. Compared to traditional manually designed features, deep learning techniques enable the learning of rich grasp representations from images, capturing the differences between various target objects and improving detection accuracy. The strengths of deep learning can significantly enhance the performance of robotic arms in unstructured environments [5,6]. Lenz et al. proposed a two-stage system with two neural networks for grasp detection, where the first network generates candidate grasp boxes and the second ranks and filters these candidates to select the optimal grasp rectangle [7]. Kumra et al. introduced a multimodal grasp detection network that integrates deep convolutional neural networks (CNNs) with support vector machines (SVMs) [8]. Mahler used the DexNet2.0 dataset to train a convolutional neural network that predicts the probability of a successful grasp based on depth images [9]. Zhou et al. proposed an end-to-end fully convolutional neural network for the real-time prediction of multiple grasp poses from RGB images [10]. Liu et al. addressed the challenge of recognizing stacked objects in robotic arm grasping and proposed an innovative Stacked Object Classification Network (SOCN) using the Transformer architecture. The self-attention mechanism in SOCN enhances the learning of stacked object features, improving the network’s classification stability [11]. Hu et al. developed a hybrid grasp prediction model based on CNNs and residual networks, combining the strengths of both to predict grasps for multiple objects simultaneously [12]. Zou et al. designed a learnable center mechanism to enhance the local feature capture, integrating it into a deformable convolutional neural network to accomplish robotic grasping tasks [13]. Villagomez et al. developed a grasping algorithm based on ResNet50, validating its effectiveness using the Robot Operating System (ROS) [14]. Yu et al. introduced an attention mechanism into ResNet for grasp detection, achieving high accuracy on both the Cornell and Jacquard datasets [15]. Li et al. combined YOLOv4 with CNNs, enabling the effective grasping of various objects with a mobile robotic arm [16]. Gao et al. designed a neural network based on depthwise separable convolution, achieving very high accuracy on the Cornell dataset with an inference speed of 28 ms [17]. Weng et al. developed a two-step convolutional neural network that enables the accurate grasping of irregular objects [18].

Despite the significant progress made by these deep learning-based object detection algorithms, their network structures remain relatively complex, requiring further lightweight optimization for real-world applications [19]. Consequently, anchor-free detection algorithms have been proposed to address the need for faster detection. These algorithms eliminate the need for predefined anchor boxes, simplifying network structures, reducing detection time, and improving accuracy through various optimization techniques. Among anchor-free algorithms, CornerNet is the first to adopt a single-stage detection approach, utilizing key points to detect objects by identifying and classifying the top-left and bottom-right corners instead of relying on anchor boxes for recognition and localization [20]. This approach significantly reduces network complexity. Building on CornerNet, Zhou et al. introduced ExtremeNet, which shares a similar framework but improves key point detection and grouping [21]. ExtremeNet predicts four vertices for each object and associates them with the object’s center point, integrating multiple sources of information to enhance prediction accuracy. Moreover, ExtremeNet’s use of center point grouping to verify predicted boxes mitigates the offsets caused by vertex predictions, further improving detection speed. As a result, ExtremeNet outperforms CornerNet in both accuracy and speed. CenterNet, another anchor-free detection algorithm, was developed as an extension of CornerNet [22]. It reformulates object detection as a center key point estimation problem, using a heatmap of the object’s center point for prediction and classification. Unlike CornerNet, which requires two key points, CenterNet only needs one, significantly improving detection speed. When using ResNet50 as the backbone, CenterNet’s detection speed can increase by 4 to 5 times under the same conditions.

Building on the CenterNet object detection network, this study introduces an enhanced grasp detection model, G-RCenterNet. This model optimizes the grasp detection algorithm in terms of network architecture, loss function, and training strategy to accurately predict the grasp box for target objects. A convolutional block attention mechanism is incorporated into the network, with channel-domain optimization in the attention module to improve detection accuracy. To meet real-time detection demands, a novel convolutional approach is adopted, increasing detection speed without compromising model precision. Furthermore, the proposed G-RCenterNet algorithm is integrated into a robotic arm grasping system, where a structured light depth camera captures target images, and the grasp detection network predicts the optimal grasp box. The contributions of this research are summarized as follows:

1. AG-RCenterNet based on the CenterNet framework is proposed for industrial robotic arm grasping tasks, providing precise grasp box predictions through image analysis and improving grasp detection effectiveness.
2. An efficient search strategy is introduced to enhance the channel and spatial attention mechanisms, significantly improving the network’s feature extraction capability and detection accuracy. This optimization allows for more flexible and precise attention to relevant features.
3. An adaptive loss function is designed specifically for grasp detection tasks, enabling the model to effectively predict feasible grasp boxes and improving localization performance and overall detection accuracy.
4. The GSConv module is introduced in the prediction decoding phase to accelerate inference speed, ensuring real-time detection without compromising accuracy.

## 2. CenterNet Algorithm

The CenterNet model extracts key information from input images using a convolutional neural network, outputting this feature information as a heatmap. By predicting on this feature map, CenterNet identifies the target’s central key points and bounding box dimensions [23]. The prediction principles for input images are as follows:

Given an input image $I\in R^{W\times H\times 3}$ with dimensions W×H×3 where *W* represents the width, *H* means the height, and 3 indicates the RGB color channels, the network aims to generate a heatmap of central key points, with each key point corresponding to a particular object class. The generated center points are denoted as $\hat{Y}\in {\left[0,1\right]}^{\frac{H}{R}\times \frac{W}{R}\times C}$, where *R* represents the output stride, and *C* is the number of object classes. In this heatmap, each key point’s position indicates the center of an object class. A predicted heatmap value of ${\hat{Y}}_{x,y,c}=1$ signals the detection of an object at that location, while a value of ${\hat{Y}}_{x,y,c}=0$ is considered the background.

Assuming $x_{1}^{\left(k\right)}$, $x_{2}^{\left(k\right)}$, $y_{1}^{\left(k\right)}$ and $y_{2}^{\left(k\right)}$ are the bounding box coordinates for the *k*th target of class $c_{k}$, the center point coordinates are represented as $p_{k}=\left(\frac{x_{1}^{\left(k\right)}+x_{2}^{\left(k\right)}}{2},\frac{y_{1}^{\left(k\right)}+y_{2}^{\left(k\right)}}{2}\right)$.

The key point estimator $\hat{Y}$ was used to predict all center points. For each object, its size $s_{k}=\left(x_{2}^{\left(k\right)}-x_{1}^{\left(k\right)},y_{2}^{\left(k\right)}-y_{1}^{\left(k\right)}\right)$ is regressed. To reduce computational complexity, a single size prediction was used for all object classes. The key point estimator predicts the center points of all targets, and, for each detected target, its size is further regressed, expressed as $s_{k}=\left(x_{2}^{\left(k\right)}-x_{1}^{\left(k\right)},y_{2}^{\left(k\right)}-y_{1}^{\left(k\right)}\right)$, where $\left(x_{1}^{\left(k\right)},y_{1}^{\left(k\right)}\right)$ and $\left(x_{2}^{\left(k\right)},y_{2}^{\left(k\right)}\right)$ represent the coordinates of the top-left and bottom-right corners of the bounding box, respectively. CenterNet is capable of predicting key point $\hat{Y}$, center point offset $\hat{O}$, and size $\hat{S}$ information. The key points provide the object’s center coordinates, and the size information includes the width and height. Thus, the network generated *C* + 4 prediction outputs at each location.

In the inference phase, to identify object classes and locate their center points, peak values in the heatmap were extracted for each class. A 3 × 3 matrix window was used to search for peaks, requiring each peak to be greater than or equal to its eight neighboring regions to ensure that local maxima were identified. The top 100 largest peaks were then selected, denoted as set $\hat{\varphi }={\left\{\left({\hat{x}}_{i},{\hat{y}}_{i}\right)\right\}}_{i=1}^{n}$, and $\left(x_{i},y_{i}\right)$ represents the coordinates of the center points. These center points’ values served as confidence measures, indicating the likelihood of the object’s center point at that location. Finally, the predicted bounding box coordinates are given by Equation (1).

(1) $$\left({\hat{x}}_{i}+\delta {\hat{x}}_{i}-{\hat{\omega }}_{i}/2,{\hat{y}}_{i}+\delta {\hat{y}}_{i}-{\hat{h}}_{i}/2,{\hat{x}}_{i}+\delta {\hat{x}}_{i}-{\hat{\omega }}_{i}/2,{\hat{y}}_{i}+\delta {\hat{y}}_{i}-{\hat{h}}_{i}/2\right)$$

where $\left(\delta {\hat{x}}_{i},\delta {\hat{y}}_{i}\right)={\hat{O}}_{{\hat{x}}_{i},{\hat{y}}_{i}}$ is the predicted position offset, and $\left({\hat{\omega }}_{i},{\hat{h}}_{i}\right)={\hat{S}}_{{\hat{x}}_{i},{\hat{y}}_{i}}$ is the predicted size.

## 3. Grasp-Reinforced CenterNet

### 3.1. Network Design

**Figure 1:**
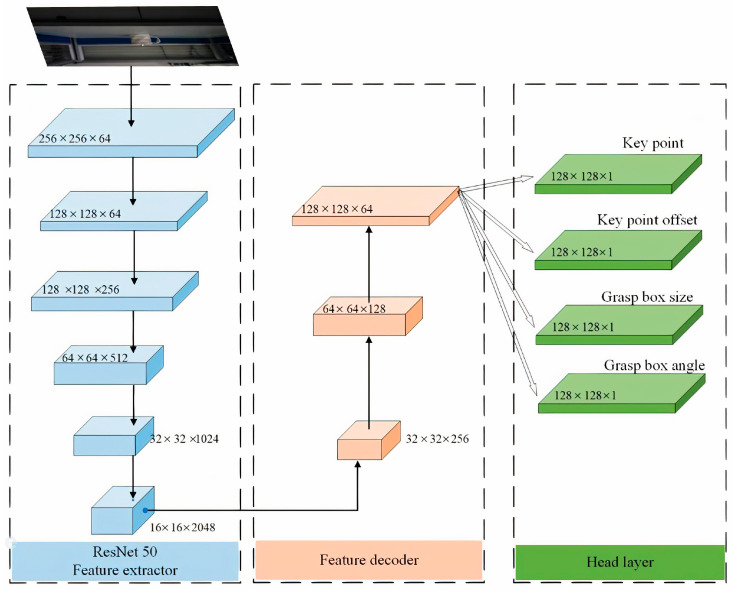
Network parameters of G-RCenterNet.

The proposed G-RCenterNet is a three-layer network model comprising a feature extraction layer, a feature decoding layer, and a head layer. Given the high demands for detection speed in practical applications, ResNet50 was selected as the feature extraction backbone. ResNet50 efficiently extracted key features from the input image and output a feature map of size 16 × 16 × 2048. Following the feature extraction network, the feature decoding layer decoded the extracted features. This layer employed three transposed convolution upsampling operations, generating a feature map of size 128 × 128 × 64, which helped restore the spatial resolution of the features and provided more precise information for the subsequent grasp box localization. The head layer, which forms the core of the network, is responsible for generating the final grasp box based on the decoded feature map. The overall framework of the proposed grasp detection network model is illustrated in Figure 1.

In the reinforced network model, four independent channels were connected after the output feature map. The key point detection channel identified key grasp points in the image, with a channel number of 1. The key point offset prediction channel estimated the displacement of each key point along the *x* and *y* axes for more accurate grasp point localization, with 2 channels dedicated to this task. The grasp box size prediction channel is responsible for estimating the width (*w*) and height (*h*) of the grasp box, determining its size, also using 2 channels. The grasp angle prediction channel predicted the grasp angle θ of the grasp box, which optimizes the object’s pose during grasping, with 1 channel allocated for this purpose. Through the outputs of these four channels, reliable predictions of the grasp box were obtained, providing a solid basis for robotic arm grasping operations.

During the prediction phase, to locate the center of the target object, heatmap peaks must be extracted for each target. This process utilizes a 3 × 3 matrix window to search for peaks, comparing the confidence score of each point on the grasp heatmap with its eight neighboring points. If a point’s confidence is higher than all its neighbors, it is considered a local peak. The entire feature map was then traversed, and the local peaks were ranked by confidence, with the top 100 peak points retained. This set of peak points is denoted as $\hat{\varphi }={\left\{\left({\hat{x}}_{i},{\hat{y}}_{i}\right)\right\}}_{i=1}^{n}$, where $\left(x_{i},y_{i}\right)$ represents the coordinates of the center points. These center points serve as confidence metrics, indicating the certainty of a target’s center at that location. In this study, the network not only identified the key points $\hat{Y}$ of the target but also predicted each key point’s position offsets $\delta_{x}$ and $\delta_{y}$, the dimensions of the grasp box (width *w* and height *h*), and the grasp angle θ. Based on these predicted key points, their offsets, the grasp box size, and the grasp angle, the output coordinates of the predicted grasp box are given as shown in Equation (2).

(2) $$\hat{k}=\left({\hat{x}}_{i}+\delta {\hat{x}}_{i},{\hat{y}}_{i}+\delta {\hat{y}}_{i},{\hat{w}}_{i},{\hat{h}}_{i},{\hat{\theta }}_{i}\right)$$

Since each key point generates only one predicted grasp detection box, the improved G-RCenterNet grasp detection algorithm does not produce multiple overlapping boxes for the same target object. Therefore, no non-maximum suppression is required in post-processing to remove redundant predictions.

### 3.2. Loss Function Design

#### 3.2.1. Key Point Estimation and Loss

Given an input image $I\in R^{W\times H\times 3}$ of size W×H×3, where *W* denotes the width, *H* the height, and 3 represents the RGB color channels, the network aims to generate a heatmap of center key points. Each key point corresponds to a specific object class, and the predicted center key point is denoted as $\hat{Y}\in {\left[0,1\right]}^{\frac{H}{R}\times \frac{W}{R}\times C}$. Here, *R* represents the down-sampling stride, and *C*, which typically denotes the number of key point types, is set to 1 in this grasp detection algorithm to indicate a feasible grasp. A predicted value of ${\hat{Y}}_{x,y,c}=1$ indicates that the target is detected, while ${\hat{Y}}_{x,y,c}=0$ signifies the background.

During training, the ground truth key points for each target in the input image are represented as $p\in R^{2}$, and their coordinates after down-sampling are transformed into $\tilde{p}=\left[\frac{p}{R}\right]$. These key points are then mapped onto the heatmap $\hat{Y}\in {\left[0,1\right]}^{\frac{H}{R}\times \frac{W}{R}\times C}$ using a Gaussian kernel $Y_{xyc}=\exp\left(-\frac{{\left(x-{\tilde{p}}_{x}\right)}^{2}+{\left(y-{\tilde{p}}_{y}\right)}^{2}}{2\sigma_{p}^{2}}\right)$, where $\sigma_{p}$ is the target size-adaptive standard deviation and $\left({\tilde{p}}_{x},{\tilde{p}}_{y}\right)$ represents the coordinates on the low-resolution feature map. In cases where Gaussian kernels for two identical target types overlap, the larger value is selected. The key point loss function is calculated as shown in Equation (3).

(3) $$L_{k}=\frac{-1}{N}\sum_{xyc}\left\{\begin{array}{l} {\left(1-{\hat{Y}}_{xyc}\right)}^{\alpha }\log{\hat{Y}}_{xyc},Y_{xyc}=1\\ {\left(1-Y_{xyc}\right)}^{\beta }{\left({\hat{Y}}_{xyc}\right)}^{\alpha }\log\left(1-{\hat{Y}}_{xyc}\right),\;\mathrm{Others} \end{array}\right.$$

where α and β are hyperparameters, *Y* represents the true key points, and *N* is the number of key points in image *I*.

#### 3.2.2. Key Point Offset and Loss

The down-sampling process during image processing can result in quantization steps that cause the center of the true bounding box to shift when mapped onto the feature map as a key point. To correct this quantization-induced displacement and ensure accurate grasp detection, the key point positions must be adjusted. This adjustment typically involves calculating the precise coordinates of the target key point, reflecting the object’s actual position and orientation and providing reliable information for subsequent grasping operations. Therefore, the key point offset issue caused by down-sampling and quantization must be fully considered when designing the grasp detection algorithm. The predicted key point offset is denoted as $\hat{O}\in P^{\frac{H}{R}\times \frac{W}{R}\times 2}$, and the offset loss is calculated using the $L_{1}$ loss function, as shown in Equation (4).

(4) $$L_{off}=\frac{1}{N}\sum_{p}\left|{\hat{O}}_{p}-\frac{p}{R}-\tilde{p}\right|$$

where *p* represents the coordinates of the center key point, and *N* is the number of positive samples.

#### 3.2.3. Grasp Box Size Prediction and Loss

As the grasp detection box is not a horizontal rectangle, it is represented by the coordinates of four corner points. Assuming these four corner points $\left(x_{1}^{k},y_{1}^{k}\right)$, $\left(x_{2}^{k},y_{2}^{k}\right)$, $\left(x_{3}^{k},y_{3}^{k}\right)$, and $\left(x_{4}^{k},y_{4}^{k}\right)$ correspond to the four corners of target *K*, the center of this irregular rectangle is determined by averaging all the *x*-coordinates and *y*-coordinates. This can be achieved by summing the four *x*-coordinates and four *y*-coordinates, then dividing by 4. Thus, the center point coordinates of target *K* can be expressed by Equation (5).

(5) $$p_{k}=\left(\frac{x_{1}^{k}+x_{2}^{k}+x_{3}^{k}+x_{4}^{k}}{2},\frac{y_{1}^{k}+y_{2}^{k}+y_{3}^{k}+y_{4}^{k}}{2}\right)$$

Similarly, the width *W* and height *H* of the grasp detection box can be described by Equations (6) and (7), respectively, and the size of the grasp detection box is given in Equation (8).

(6) $$W=\sqrt{{\left(x_{2}^{k}-x_{1}^{k}\right)}^{2}+{\left(y_{2}^{k}-y_{1}^{k}\right)}^{2}}$$

(7) $$H==\sqrt{{\left(x_{2}^{k}-x_{3}^{k}\right)}^{2}+{\left(y_{2}^{k}-y_{3}^{k}\right)}^{2}}$$

(8) $$s_{k}=\left(\sqrt{{\left(x_{2}^{k}-x_{1}^{k}\right)}^{2}+{\left(y_{2}^{k}-y_{1}^{k}\right)}^{2}},\sqrt{{\left(x_{2}^{k}-x_{3}^{k}\right)}^{2}+{\left(y_{2}^{k}-y_{3}^{k}\right)}^{2}}\right)$$

The size prediction is evaluated using the *L*_1_-loss function, as shown in Equation (9).

(9) $$L_{size}=\frac{1}{N}\sum_{k=1}^{N}\left|{\hat{S}}_{pk}-s_{k}\right|$$

where ${\hat{S}}_{pk}$ represents the reference grasp box size for target *k*, and *N* is the number of positive samples.

#### 3.2.4. Grasp Angle Prediction and Loss

Predicting the grasp angle θ is a critical step in the grasp detection algorithm. The proposed method defines the grasp angle θ as the angle between the projection of the robotic gripper’s jaws on the image plane and the horizontal direction of the image. This angle ranges from 0 to 180 degrees, as the end-effector of the LBR LM3 robotic arm used in this study can rotate clockwise and counterclockwise in the horizontal plane, accommodating objects placed in various orientations. In the 2D image plane, the grasp box is represented by its four corner points, and the grasp angle θ is defined as the direction of the line connecting the first two corner points. This method can also be applied to estimate the grasp angle for the custom grasp detection dataset.

The grasp angle is computed through the following steps:

(1) Identify the coordinates of the first two corner points, denoted as $\left(x_{1},y_{1}\right)$ and $\left(x_{2},y_{2}\right)$.
(2) Compute the direction vector by subtracting the coordinates of the first point from the second point, yielding $\left(\Delta x,\Delta y\right)=\left(x_{2}-x_{1},y_{2}-y_{1}\right)$.
(3) Calculate the grasp angle θ using the arctangent function $\mathrm{atan}2\left(\Delta y,\Delta x\right)$. The *atan2* function returns the angle formed by the vector $\left(\Delta x,\Delta y\right)$ and the positive direction of the *x*-axis, with a range of $\left(-\pi ,\pi \right)$. Since the grasp angle θ ranges from $\left(0,\pi \right)$, the result of *atan2* is adjusted accordingly.

The adjusted angle θ is calculated by Equation (10).

(10) $${\theta =\tan}^{-1}2(\Delta y,\Delta x)$$

If θ<0, it is modified to $\pi -\left|\theta \right|$ to ensure it falls within the specified range. The final grasp angle θ is expressed by Equation (11).

(11) $${\theta =\tan}^{-1}\left(\frac{y_{2}^{k}-y_{1}^{k}}{x_{2}^{k}-x_{1}^{k}}\right)$$

The angle prediction is evaluated using the *L*_1_-loss function, as shown in Equation (12).

(12) $$L_{\theta }=\frac{1}{N}\sum_{k=1}^{N}\left|{\hat{\theta }}_{pk}-\theta_{k}\right|$$

where ${\hat{\theta }}_{pk}$ represents the grasp angle of target *k*, and *N* is the number of positive samples.

#### 3.2.5. Total Loss Function

In computing the loss functions, target sizes are not normalized but instead calculated using raw pixel coordinates to preserve data consistency and authenticity. However, this approach may result in larger loss values, which could negatively affect model training and convergence speed. To balance the contributions of different loss functions during training, appropriate weights are assigned to each loss term. The total loss function is given by Equation (13).

(13) $$L_{\det}=L_{k}+\mu_{off}L_{off}+\mu_{size}L_{size}+\mu_{\theta }L_{\theta }$$

By properly adjusting these weights, each loss function can contribute effectively during training, improving the model’s performance and stability.

### 3.3. Improved Convolution Block Attention Module

**Figure 2:**
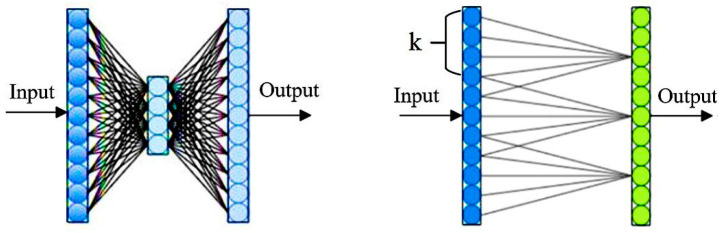
Two search mechanisms.

Building upon the convolution block attention module (CBAM), this study introduces a more objective and efficient search strategy to optimize the channel domain within the module, enhancing both its performance and efficiency [24]. Specifically, a one-dimensional convolution operation was employed to replace the traditional fully connected layers in the shared network for processing input parameters. This design captures information from the neighboring regions of neuron nodes within the convolutional structure, reducing the loss of target feature information during dimensionality reduction. Through successive convolution processes, the neurons continuously learn feature information, enriching the model’s ability to express target features. This method optimizes the network structure and improves its efficiency in processing target features. As illustrated in Figure 2, the left panel shows the traditional fully connected layer-based search mechanism, while the right panel presents the more efficient channel search method proposed in this paper.

In Figure 2, the variable *k* represents the size of the one-dimensional convolution kernel, reflecting the breadth of the channel. As shown in Figure 2, the proposed channel attention mechanism enhances feature learning by enabling interactions between selected channels and neighboring channels. This cross-channel interaction allows feature information to flow across different channels. With the continuation of convolution operations, the neurons acquire increasingly comprehensive and enriched feature information, thereby improving the network’s ability to represent target features. Importantly, the proposed channel attention method enhances the utilization of channel information without increasing the model’s complexity, thereby boosting the overall performance of the network. The formula for calculating the convolution kernel k is shown in Equation (14).

(14) $$k=\psi \left(C\right)=\left|\frac{\log_{2}\left(C\right)}{\gamma }+\frac{b}{\gamma }\right|$$

where *C* denotes the number of channels in the input feature map, and γ and *b* are pre-set parameters with γ = 2 and *b* = 1. ${\left|t\right|}_{odd}$ represents the nearest odd number. Based on Equation (13), the selected adaptive k value is positively correlated with the number of channels in the feature map, meaning that, as the number of channels increases, the required *k* value also increases. Since the output dimension remains constant, the selection of adaptive values exhibits flexibility. In practical applications, the adaptive value can either be set to a fixed value or adjusted dynamically based on channel dimension variations. This flexibility allows the model to adapt to feature maps with varying channel numbers, optimizing performance accordingly.

After implementing these improvements to the channel search mechanism, the enhanced CBAM is integrated into the CenterNet object detection network. To ensure that the inclusion of the attention mechanism does not alter the ResNet50 architecture, the attention mechanism is added to the first and last convolutional layers of the network.

### 3.4. Lightweight Method Based on Improved Convolutional Modules

**Figure 3:**
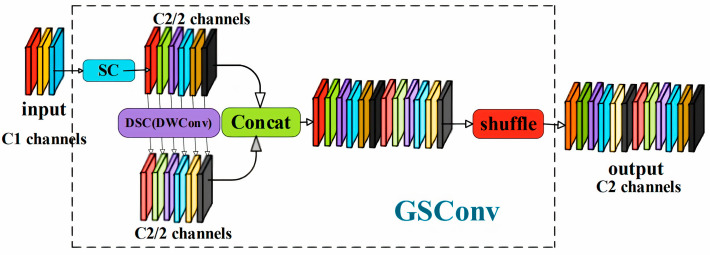
GSConv module structure.

Most methods for constructing lightweight network models primarily focus on replacing standard convolutions with a large number of depthwise separable convolutions. However, this approach often leads to a loss of semantic information, which reduces the accuracy of the predictions. Therefore, there is a need for a new approach that can reduce the number of parameters without compromising model accuracy. The GSConv module [25], a novel convolutional method, addresses this challenge. The structure of the GSConv module is illustrated in Figure 3.

As shown in Figure 3, the convolutional module consists of three operations: convolutional layers, batch normalization, and activation layers. SC refers to channel-dense convolution, DSC denotes channel-sparse convolution, and DWConv represents the DSC operation [26]. The GSConv module first performed down-sampling using a standard convolution, followed by a depthwise convolution using DWConv. The results from these two convolutions were concatenated, and a shuffle operation [27] was then applied to rearrange the channels, bringing together corresponding channels from the previous convolutions. These steps ensure that the output of the channel-sparse convolution closely approximates that of the channel-dense convolution, thereby minimizing the loss of semantic information. Additionally, this approach simplifies the convolutional operations, reducing the computational load of the neural network.

To further accelerate prediction calculations, feedback images in the neural network undergo a series of transformations within the backbone network. This process primarily involves the transfer of spatial information to the channel domain. During this process, the inevitable compression of the spatial dimensions (width and height) and the expansion of the channel dimensions can lead to some loss of semantic information. To mitigate this, channel-dense convolution was introduced to preserve hidden connections between channels, maintaining the integrity of the information. In contrast, channel-sparse convolution completely severs these connections, potentially leading to information bottlenecks and degraded model performance. Therefore, when designing neural networks, a balance must be struck between these two types of convolutions to accelerate computation while maintaining high model performance. In CenterNet object detection networks, transposed convolution and normalization operations were employed during output to compress the spatial information of feature maps.

Inspired by the GSConv module, this module was incorporated into the network’s decoding process to retain as much semantic information as possible. The GSConv module maintains efficient inter-channel connections with low time complexity, allowing the network to effectively preserve the flow and interaction of information while ensuring computational efficiency. This enhances the model’s expressive and generalization capabilities. Due to the improved non-linear expressiveness afforded by the DSC layer (depthwise separable convolution) and the shuffle operation, the GSConv module offers significant advantages in lightweight detectors. The DSC layer design effectively reduces computational complexity and the number of parameters while maintaining high performance, unlocking the GSConv module’s potential in lightweight detectors. The shuffle operation further strengthens the network’s non-linear characteristics, improving its generalization and robustness. Therefore, the standard upsampling convolution modules in the original CenterNet code were replaced with GSConv modules, leading to faster inference speeds.

**Figure 4:**
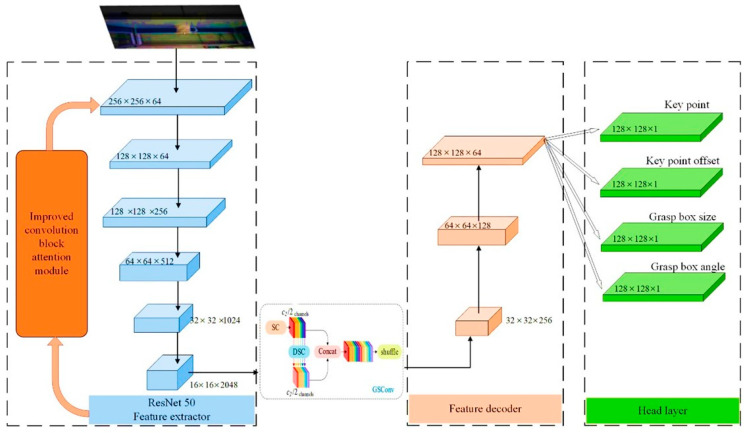
G-RCenterNet grasp target detection network model.

Improvements to the designed grasp target detection network include incorporating attention mechanisms and replacing the network’s upsampling convolution modules. The improved G-RCenterNet grasp target detection network model is shown in Figure 4.

## 4. Experimental Validation

### 4.1. Grasp Detection Dataset

Accurately obtaining the pose information of target objects is critical for robotic arms to perform grasping tasks successfully. To preserve the completeness and accuracy of key object information and ensure the success and reliability of the grasping task, this study adopts a five-parameter representation method for grasping rectangles in a two-dimensional plane [28]. This approach uses five parameters to describe the grasp: *x* and *y* represent the coordinates of the grasp point in the image coordinate system, determining the location of the grasp point in 2D space. θ represents the angle between the gripper’s opening direction and the horizontal axis of the image coordinate system, describing the gripper’s orientation. The width (*w*) and height (*h*) represent the grasp width and length of the gripper in the image plane, respectively, defining the gripper’s opening size. These five parameters together form a complete 2D grasp rectangle description. By using coordinate transformations and projections, this 2D grasp rectangle can be mapped into 3D space to obtain the object’s pose in the robotic arm’s coordinate system. This mapping preserves the key features of the grasp rectangle, allowing the robotic arm to execute precise grasping operations in 3D space. The grasp rectangle is mathematically represented as Equation (15).

(15) $$K=\left(x,y,h,w,\theta \right)$$

where *x* and *y* are the grasp point coordinates in the image coordinate system, θ describes the angle between the gripper’s opening and the horizontal axis, and *w* and *h* denote the grasp width and length in the image plane.

During the grasp detection phase, the grasp detection network processes input data (such as RGB and depth images) to predict the grasp rectangle (g). This grasp rectangle, expressed in 2D space, includes information such as the grasp point’s 2D coordinates, the rectangle’s size, and its orientation [29]. To effectively apply this 2D information to 3D robotic arm grasping operations, camera calibration and hand–eye calibration results are utilized to convert the grasp rectangle into the robotic arm’s end-effector pose.

The transformation from the 2D grasp rectangle in the image to the robotic arm’s grasp pose in 3D space was calculated using Equation (16). In this equation, $T_{r}$ was determined by hand–eye calibration, representing the transformation between the image space and the robotic arm’s operational space. $T_{d}$ was derived from camera calibration and represents the mapping from pixel coordinates to the camera coordinate system. $G_{i}$ denotes the grasp rectangle in the image.

(16) $$G=\left(T_{r}\left(T_{d}G_{i}\right)\right)$$

An RGB camera was employed to capture the object’s planar image, and a grasp detection algorithm predicts the grasp rectangle, yielding the 2D grasp coordinates. Depth information was then obtained via a depth camera, and the target object’s 3D grasp pose was computed through a series of coordinate transformations.

The G-RCenterNet algorithm estimates the target based on central key points, which necessitates robust feature information from central points across different perspectives during training. This is strongly dependent on a diverse and sufficiently large dataset. The diversity and richness of the dataset directly determine the model’s performance ceiling, while a strong algorithm helps the model approach this performance limit. However, in practical applications, obtaining sufficient target data samples is often challenging. To address this issue, data augmentation techniques were widely applied to increase the diversity and richness of the dataset, preventing the model from overfitting during training.

In this study, two datasets were used to validate and evaluate the effectiveness of the grasp detection algorithm: the widely used Cornell grasping dataset and a custom-made dataset. The Cornell grasping dataset is a standard benchmark for evaluating grasp detection networks, providing a widely accepted platform for model performance evaluation. The custom dataset was created to conduct grasp trials in real-world environments, closely aligning with the practical application scenarios faced by robotic arms. By using both datasets, a more comprehensive exploration of the algorithm’s performance and applicability was conducted.

**Figure 5:**
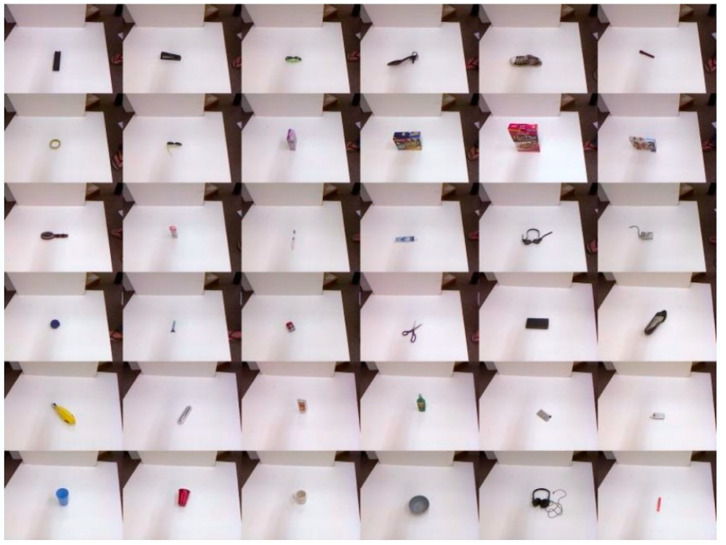
Cornell Grasp Dataset.

**Figure 6:**
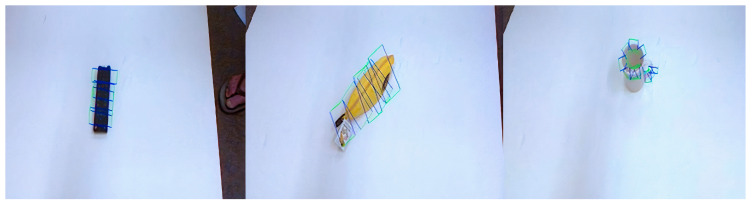
Examples of feasible grasps.

**Figure 7:**
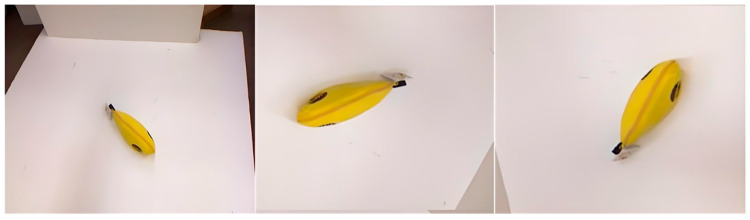
Data augmentation.

The Cornell grasping dataset, open-sourced by Cornell University, is a specialized dataset for evaluating grasp detection models. Initially, it contained 885 image samples, each accompanied by RGB images, depth images, point cloud files, and positive and negative grasp labels. After augmentation, the dataset expanded to 1035 images, covering 244 different objects. Figure 5 shows some sample objects from the Cornell Grasping Dataset. Only positive sample labels were used for training in this study, and Figure 6 illustrates a feasible grasp example. Upon obtaining the Cornell grasping dataset, preprocessing was required. This involves two primary tasks: dataset expansion and quality improvement. Neural networks often rely on a large number of training samples to fully learn and extract object features. However, the limited image quantity in the Cornell dataset might lead to suboptimal training outcomes and insufficient generalization. To address this, data augmentation techniques—such as rotation, translation, and scaling—were applied to the original images, increasing the dataset’s size. Additionally, sharpening and blurring methods were used to enhance image quality. By cropping the images, background noise was minimized, further improving the dataset quality. Figure 7 shows augmented images, where the original image is center-cropped and then subjected to random rotations and translations to generate multiple variants.

**Figure 8:**
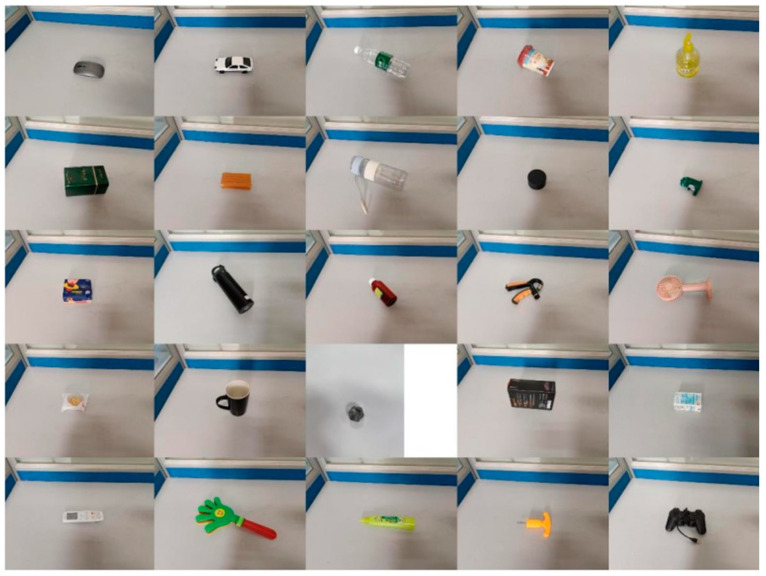
Custom-made dataset.

This study focuses on the detection and grasping of target objects in real-world environments. To achieve this, a custom grasp detection dataset was created, as shown in Figure 8. The dataset consists of 300 images of commonly encountered objects such as cups, paper boxes, and nuts, with the goal of testing robotic arm performance in practical grasping tasks. Images were captured using a depth camera, obtaining both RGB and depth information of the target objects. Data augmentation was performed on the custom dataset following the same procedure used for the Cornell dataset.

The collected images were annotated using the LabelImg2 (version 2.13.0) software, which allows the use of rotated bounding boxes for object annotation. Multiple feasible grasp boxes were labeled for each target. The annotation results are saved in XML files, which recorded the center point, width, height, and rotation angle of the boxes. The rotation angle is defined with 0 degrees corresponding to the 12 o’clock position, with a range of 0 to 360 degrees.

### 4.2. G-RCenterNet Training

The experiments were conducted on a Linux-based platform with hardware specifications including a 12 vCPU Intel(R) Xeon(R) Platinum 8255C CPU @ 2.50 GHz, 16 GB RAM, and an NVIDIA GeForce RTX 2080Ti GPU (manufactured by NVIDIA Corporation, Santa Clara, CA, USA). The training was implemented using the PyTorch deep learning framework (developed by Meta Platforms, Beijing, China) with Python 3.7 as the programming language. The training strategy on the dataset is as follows:

The improved G-RCenterNet algorithm is trained on the Cornell Grasp Dataset. The Adam optimizer is used for training, with key parameters set as follows: batch size of 8, an initial learning rate of 0.001, and a total of 200 training epochs. Validation was conducted on the custom dataset.

### 4.3. Experimental Results and Analysis

The performance of the proposed grasp detection network is evaluated on the Cornell Grasp Dataset using the metrics proposed by Sulabh Kumra et al. A predicted grasp is considered correct if it met two conditions:

(1) The difference in rotation angle between the predicted and ground truth grasp boxes is within 30°.
(2) The Jaccard index between the predicted and ground truth grasp boxes is greater than 25%.

The Jaccard index, as defined in Equation (17).

(17) $$J\left(G,\hat{G}\right)=\frac{G\cap \hat{G}}{G\cup \hat{G}}$$

where G is the area of the predicted grab box, and $\hat{G}$ is the area of the actual grab box. $G\cap \hat{G}$ is the intersection of the two rectangular boxes. $G\cup \hat{G}$ is the union of the two rectangular boxes.

**Figure 9:**
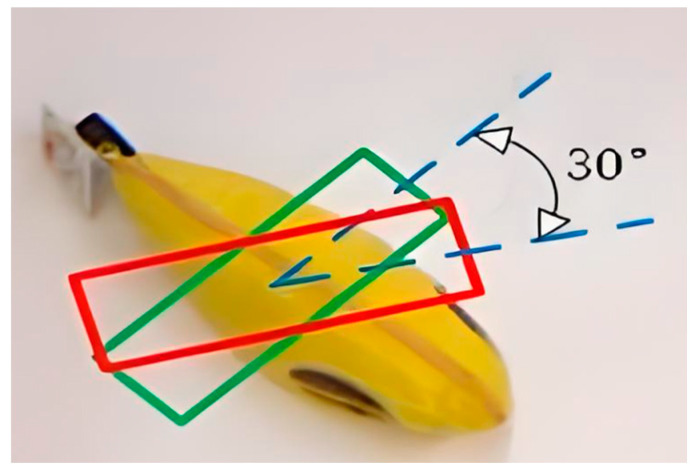
Predicted grab box and true grab box.

**Figure 10:**
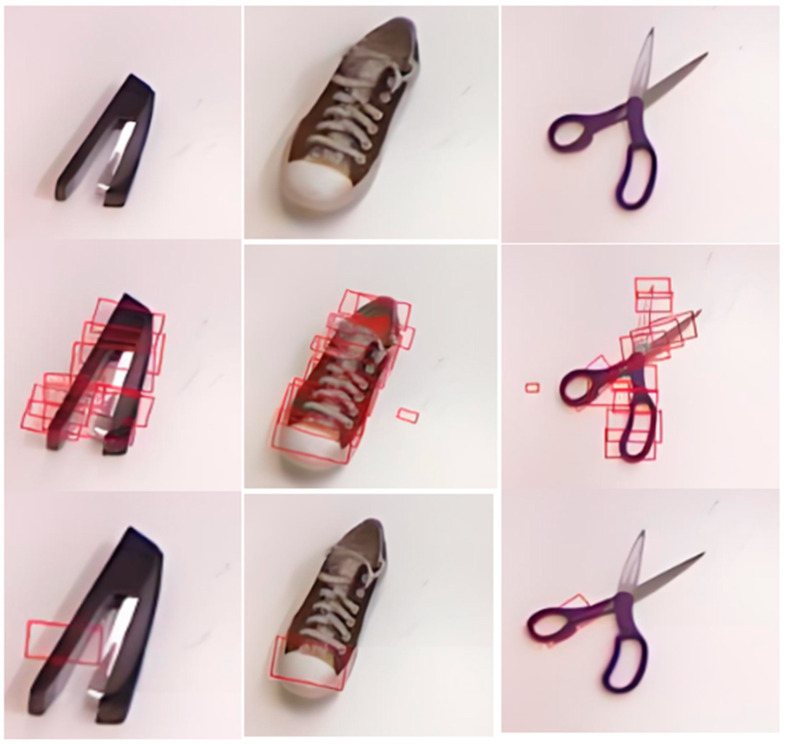
Cornell dataset detection results.

Predicted grab box and true grab box is presented in Figure 9. The red box is the predicted grad box and the blue box is the true grab box. The grasp detection network is validated on the Cornell Grasp Dataset, achieving a detection accuracy of 95.6% with the improved G-RCenterNet algorithm. Figure 10 shows sample detection results on the test set of the Cornell dataset. The first row contains target objects from the dataset, while the second row shows the predicted grasp box output by the detection model, generated based on heatmap peak points, positional offsets, grasp box dimensions, and grasp angles. The third row presents the best predicted grasp, generated using the highest confidence peak point and its corresponding features. These results demonstrate the model’s ability to accurately learn the feasible grasp characteristics and detect graspable objects within the images.

To evaluate the effectiveness of the proposed improvements, a set of ablation experiments was conducted. The results, shown in Table 1, compare the performance of the algorithm before and after the improvements under the same experimental conditions.

As shown in Table 1, the improvements made in this study enhanced the detection accuracy and speed of the network model. The convolutional block attention mechanism boosts detection accuracy but slightly reduces the detection speed. The efficient channel search mechanism helps mitigate some of the speed loss, while the GSConv convolution module compensates for the detection speed reduction caused by the attention module. By leveraging the strengths of both methods through careful design, the G-CenterNet grasp detection network model achieves improved detection accuracy and speed.

To further understand the advantages and limitations of the proposed method, a performance comparison with other State-of-the-Art grasp detection algorithms on the same dataset was conducted, as shown in Table 2.

ResNet18 achieves good performance in terms of speed, with 48.4 FPS, but due to its relatively shallow network structure, its feature extraction capability is weaker, resulting in an accuracy of 93.3%. This makes it slightly lower than other backbone networks, and its performance is somewhat insufficient for grasp detection tasks in complex backgrounds. MobileNetV2, as a lightweight network, achieves the highest inference speed of 41.1 FPS. However, its accuracy is lower at 91.7%, making it suitable for mobile devices and real-time applications but less effective for complex grasp detection tasks where higher accuracy is required.

The deeper network architecture of ResNet101 significantly enhances its feature extraction capability, achieving an accuracy of 96.9%, slightly higher than ResNet50. However, due to its larger computational load and parameter count, its inference speed is 68.5 FPS, which does not meet real-time requirements. The proposed method, using ResNet50, strikes a good balance between accuracy and inference speed, achieving 95.6% accuracy and 53 FPS. ResNet50 maintains high grasp detection accuracy in complex backgrounds while meeting real-time detection needs, confirming its suitability and effectiveness for this study.

**Figure 11:**
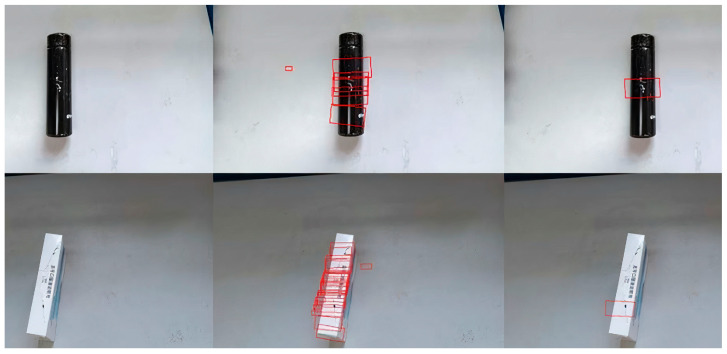
Cornell dataset detection results.

Through quantitative analysis of the performance of these models, the effectiveness and advantages of the proposed algorithm can be clearly assessed. As shown in Table 2, the improved G-CenterNet grasp detection algorithm significantly outperforms other detection algorithms in both accuracy and detection speed. It achieves a detection accuracy of 95.6% on the Cornell Grasp Dataset, with an image processing time of approximately 19 ms, meeting real-time detection requirements. The comparison results of all methods are presented in Table 3. Compared to mainstream methods such as Multi-grasp ResNet-50, GR-ConvNet-RGB-D, and SE-ResNet, G-RCenterNet not only maintains a high accuracy but also further improves detection speed. Notably, in comparison to the more accurate GR-ConvNet-RGB-D (96.7%) and SE-ResNet (97.1%), G-RCenterNet reaches an inference speed of 53 FPS, outperforming both methods in terms of real-time performance. Overall, G-RCenterNet achieves a good balance between accuracy and speed, making it suitable for real-time grasp detection tasks in practical applications. The detection results on our custom dataset are shown in Figure 11.

The accuracy on the custom dataset is 90.7%, slightly lower than on the Cornell dataset. This discrepancy is attributed to the custom dataset being used only for validation, where the grasp detection network did not fully learn all target features. Through the carefully designed network architecture and training strategy, the G-RCenterNet grasp detection network achieves high detection accuracy while maintaining fast inference, providing strong support for subsequent robotic arm grasping tasks.

## 5. Robotic Arm Grasping Experiments in the Real World

**Figure 12:**
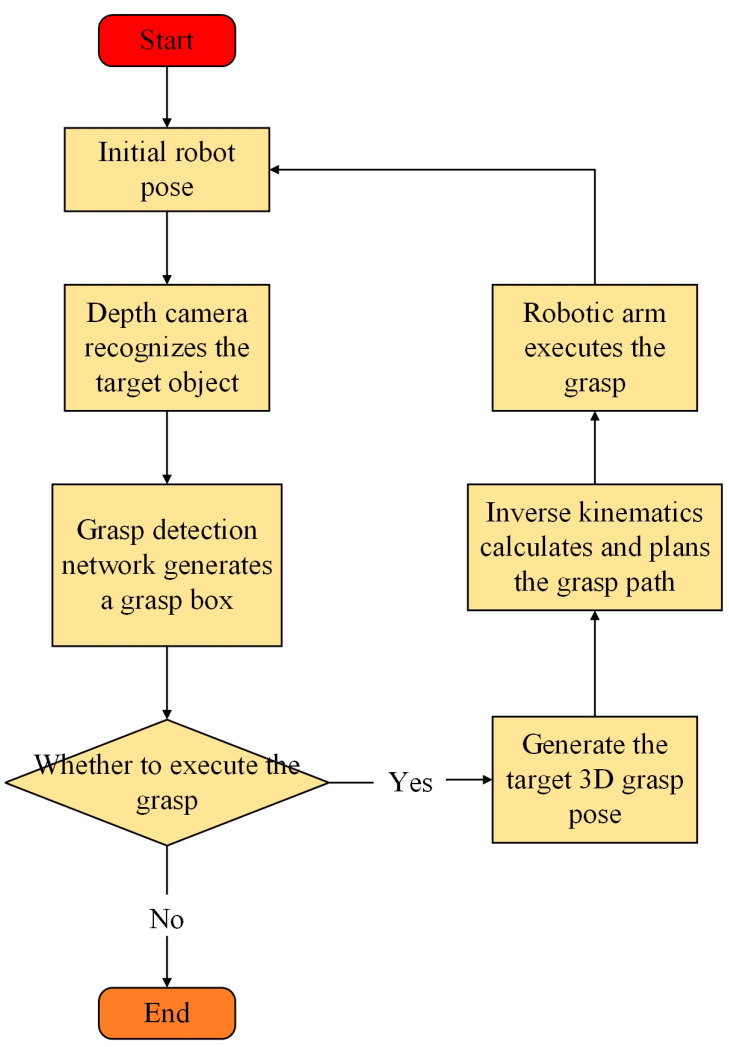
Grasping process flowchart.

In the robotic arm grasping experiments, this study focused on grasping operations involving target objects in real-world scenarios. During autonomous grasping tasks, the robotic arm system first utilized a depth camera to obtain the depth information of the target object, enabling the identification of its geometric shape and spatial position. The grasp detection module employed the G-RCenterNet grasp detection network model to process the RGB images of the identified target object, predicting the 2D grasp box coordinates. By fusing the 2D grasp box coordinates with the depth information, the 3D grasp pose coordinates of the target object in the camera coordinate system were obtained. Subsequently, the coordinates of the target object were transformed from the camera space to the robotic arm space using matrix transformation relations. After acquiring the coordinate information, inverse kinematics calculations were performed to determine the joint angles of the robotic arm, followed by motion planning algorithms to optimize the grasping path. The grasping process is illustrated in Figure 12.

During the execution of the grasping task, if the robotic arm does not detect a graspable target, the task is terminated; if a target is detected, the robotic arm proceeds to execute the grasping task and returns to its initial pose after completion.

**Figure 13:**
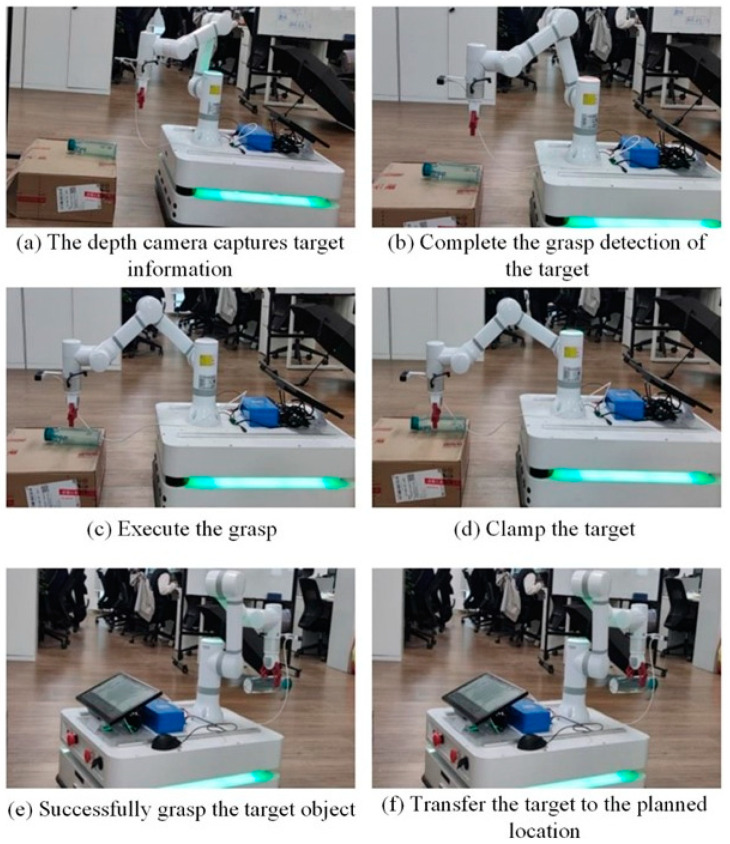
Robotic arm grasping process.

In the practical environment, robotic arm grasping experiments are conducted, maintaining the position of the robotic base relative to the moving robot throughout the grasping process. After successfully grasping the target, the robotic arm transfers it to a designated target location. Figure 13 showcases the grasping process of the robotic arm.

**Figure 14:**
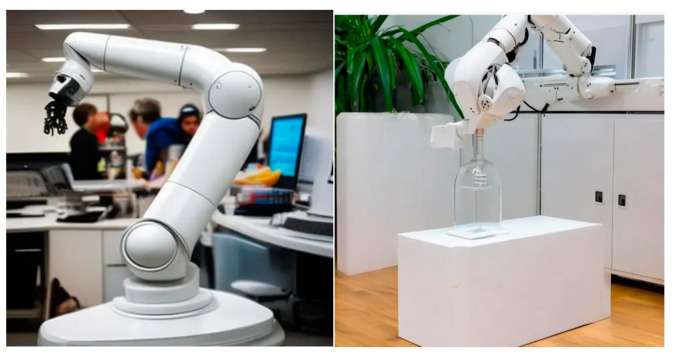
Grasping in different backgrounds.

To comprehensively evaluate the accuracy and success rate of the entire robotic arm grasping system, systematic grasping experiments were conducted in various background environments, as shown in Figure 14. These experiments aimed to test the performance of the robotic arm’s end effector in complex and dynamic environments, particularly its ability to accurately reach target points and successfully execute grasping tasks. The experiments emphasized the spatial relationship between the end effector and the target object, ensuring that the end effector could accurately reach the target point according to the predefined trajectory and pose. Additionally, stability and success rates during the grasping process were meticulously recorded and analyzed.

**Figure 15:**
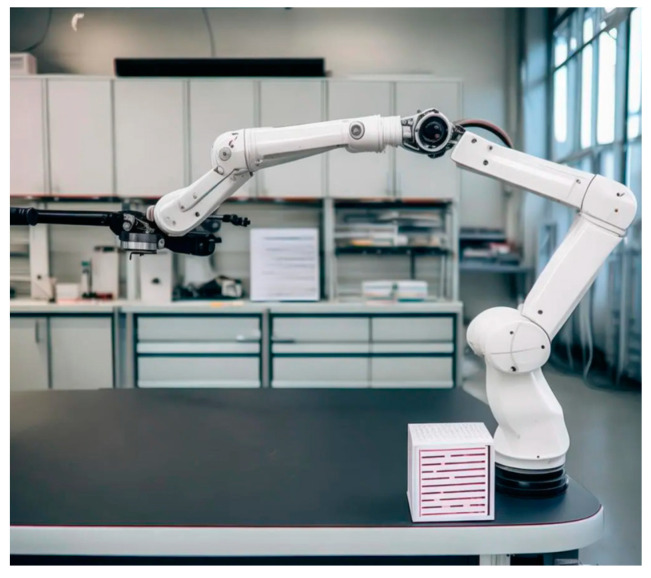
Grasping small target.

Grasping experiments are also conducted on small targets such as nuts. M6 nuts are selected for the experiments, and, as shown in Figure 15, the robotic arm successfully grasped the nut. The results of the nut grasping experiments demonstrated that the robotic arm could effectively grasp the nut, indicating that the improved grasp detection algorithm performs well even for small targets. This success can be attributed to the grasp detection network designed based on the CenterNet object detection framework, which utilizes key point detection to effectively identify the center point of the target, giving it a significant advantage for small target recognition.

To rigorously validate the accuracy and success rate of the entire robotic arm grasping system, this section designed and executed specific grasp accuracy experiments. Four representative target objects commonly found in the custom dataset were selected: plastic bottles, cups, nuts, and mice. These targets not only represent everyday items but also vary in shape, size, and texture, adding complexity and practicality to the experiments. The experimental procedure involved random placements and grasping to simulate various scenarios encountered in real-world environments. For each target object, 25 random placements and grasping attempts were conducted, totaling 100 experiments. In each trial, the performance of the robotic arm’s end effector was recorded, focusing on whether it accurately reached the target point and successfully executed the grasping task. To facilitate analysis and summarize the results, the data from these 100 experiments are compiled and presented in Table 4. This table provides a clear overview of the system’s performance under different conditions and serves as a basis for subsequent improvements and optimizations.

In the 100 grasping experiments, the robotic arm successfully completed 86 effective grasps, resulting in a success rate of 86%. Among the results, the success rate for grasping nuts is relatively low, primarily due to their smaller size, which increased the likelihood of slipping from the robotic arm’s gripper during grasping. To improve the success rate for small targets such as nuts, efforts should focus on enhancing the gripper design and adopting better grasping strategies to ensure a higher success rate when dealing with objects of varying sizes and shapes. Despite the grasp detection model accurately identifying the nut in both the grasp detection network model predictions and the grasp detection tests, the low success rate for grasping nuts is a challenge. Excluding the nut from this analysis, the robotic arm achieved a success rate of 90.7% for more common objects. This indicates that the robotic arm grasping system designed in this study demonstrates competitive success rates for typical items compared to previous research.

As robotic arm grasping technology continues to evolve, evaluating the performance of grasp detection models is no longer limited to assessing prediction accuracy on detection datasets. Instead, it increasingly relies on the success rates of real-world grasping tasks to evaluate the performance of grasp detection network models. Through these grasping experiments, the robotic arm grasping system designed in this study achieved a success rate of 90.7% for common objects, effectively demonstrating the validity of both the proposed grasp detection algorithm and the constructed robotic arm grasping system.

## 6. Conclusions

This study focuses on the problem of target grasping in unstructured environments and presents a grasp target detection algorithm called G-RCenterNet. Specifically, the grasp target detection network model utilizes ResNet50 as its backbone and incorporates a loss function tailored to the characteristics of grasp detection tasks, enabling it to output grasp boxes for target objects. The introduction of the CBAM enhances the network’s ability to extract target features. Additionally, a more efficient search strategy replaces the traditional fully connected layers in the channel attention module, improving the detection accuracy of the network model. During the prediction decoding stage, the GSConv module is integrated to accelerate the model’s inference speed. The designed grasp target detection network is trained and validated on both the Cornell Grasping Dataset and the custom dataset, achieving an accuracy of 95.6% and a detection speed of 53 FPS, meeting real-time detection requirements. A robotic arm grasping system is established, utilizing the proposed G-RCenterNet grasp target detection algorithm as the inference module to conduct robotic arm grasp detection test experiments, successfully completing the real-time grasp detection of target objects. In the grasp detection test experiments, out of 100 grasping operations, the robotic arm successfully completed 86 effective grasps, achieving a success rate of 90.7% for common objects. This validates the excellent performance of both the designed G-RCenterNet grasp target detection algorithm and the robotic arm grasping system.

Through comparative experimental analysis, it can be observed that the proposed method achieves the fastest inference speed, meeting real-time requirements. However, the grasp detection accuracy still lags behind the latest algorithms. In the future, we plan to conduct further research on deep learning-based grasp detection algorithms with the goal of developing methods that are faster, more efficient, and more accurate.

## Figures and Tables

**Table 1:** Ablation experiments with different strategies.

Network Model	Accuracy (%)	FPS
G-CenterNet	90.7	48.6
G-CenterNet + CBAM	94.4	44.8
G-CenterNet + improved CBAM	95.8	47.1
G-CenterNet + GSConv	89.9	54.1
G-CenterNet + improved CBAM + GSConv (Proposed)	95.6	53

**Table 2:** The comparison results of all methods.

Backbone Network	Accuracy (%)	FPS
ResNet18	93.3	48.4
MobileNetV2	91.7	41.1
ResNet101	96.9	68.5
ResNet50 (Proposed)	95.6	53

**Table 3:** The comparison results of all methods.

Source	Method	Accuracy (%)	FPS
Morrison [27]	GG-CNN	73	52.63
Kumra [7]	ResNet-50×2	89.2	9.71
Lenz [6]	SAE, struct. reg	73.9	0.74
Chu [30]	Multi grasp RestNet-50	96.0	8.33
Karaoguz [31]	GRPN	88.7	5
Asif [32]	GraspNet	90.2	41.67
Sulabh [33]	GR-ConvNet-RGB-D	96.7	50
Yu [15]	SE-ResNet	97.1	40
Proposed	G-RCenterNet	95.6	53

**Table 4:** Grasp experiment statistics.

Category	Grasp Attempts	Successful Grabs	Success Rate (%)
Plastic bottle	25	23	92
Cup	25	24	96
Nut	25	18	72
Mouse	25	21	84
Total	100	86	86

## Data Availability

Dataset available upon request from the authors.
